# Research progress and value of albumin-related inflammatory markers in the prognosis of non-small cell lung cancer: a review of clinical evidence

**DOI:** 10.1080/07853890.2023.2192047

**Published:** 2023-04-10

**Authors:** Chuan-long Zhang, Meng-qi Gao, Xiao-chen Jiang, Xue Pan, Xi-yuan Zhang, Yi Li, Qian Shen, Yan Chen, Bo Pang

**Affiliations:** a Guang’anmen Hospital, China Academy of Chinese Medical Sciences, Beijing, China; b Wangjing Hospital, China Academy of Chinese Medical Sciences, Beijing, China; c International Medical Department of Guang’anmen Hospital, China Academy of Chinese Medical Sciences, Beijing, China

**Keywords:** Non-small lung cancer, inflammation, albumin, markers, prognosis

## Abstract

Inflammatory markers have a wide range of predictive values in the prognosis of non-small lung cancer (NSCLC). Poor nutritional status usually means a poor prognosis in patients with NSCLC, which is widely recognized by oncologists and nutritionists. Serum albumin has a certain value in evaluating the prognosis of patients. Several inflammatory albumin-related markers have been proposed, but they have not been widely used in predicting the prognosis of NSCLC in clinical practice. We aim to systematically review the published clinical evidence of albumin-related inflammatory markers in predicting the prognosis of NSCLC and to describe their progress and value. The results showed that the markers included in the review could be prognostic indicators in patients with NSCLC. However, we found that the cut-off value of albumin-related inflammatory markers with quantitative nature was very chaotic and needed to be defined by recognized standards. We summarized and compared the advantages and disadvantages of these markers, but a prospective cohort study with long-term follow-up after adjustment for important confounders is still necessary. Whether the results and conclusions could be directly applied in clinical practice needs to be identified and evaluated. There is an urgent need to classify and standardize the albumin-related inflammatory markers that play an important role in the prognosis of NSCLC, which is the key to ensuring the transformation from clinical study to clinical application.

## Key Messages

- Albumin-related inflammatory markers could be prognostic indicators in non-small cell lung cancer.
- The classification and standardization of albumin-related inflammatory markers guarantee the transformation from clinical study to clinical application.
- Future prospective studies of albumin-related inflammatory markers excluding confounding factors are very necessary.

## Introduction

At present, lung cancer remains the leading cause of cancer-related death in humans worldwide, despite the increasing diversity of treatment therapy. Non-small lung cancer (NSCLC) accounts for about 80% of all cases [1]. We previously reported the role of the inflammatory microenvironment in lung cancer [2]. Inflammation has a broad impact on the formation and progress of NSCLC [3], including proliferation and survival of cancer cells, angiogenesis, tumour metastasis, and tumour response to chemotherapeutic drugs and hormones [4–7].

With an in-depth understanding of the relationship between the molecular mechanism of lung cancer and the inflammatory microenvironment, we found that clinical monitoring of inflammatory markers in patients with NSCLC had important clinical value for prevention and developmental control [8]. There are various laboratory markers of systemic inflammation including C-reactive protein (CRP), neutrophil (NEU), lymphocyte (LYM), and so on. CRP plays an important role in host defence mechanisms and inflammatory responses to infectious agents, mainly produced by hepatocytes in response to stimulation by interleukin-6 (IL-6), tumour necrosis factor-α (TNF-α), and interleukin-1β (IL-1β), which in turn can reactivate leukocytes and platelets, creating a cycle of action [9]. However, one single inflammatory index has limitations in independently predicting survival in patients with NSCLC. Therefore, further studies on composite prognostic indicators are very necessary. Exploring more, newer, and better composite inflammatory markers will guide improving the prognosis of patients with NSCLC in the future.

**Figure 1.**
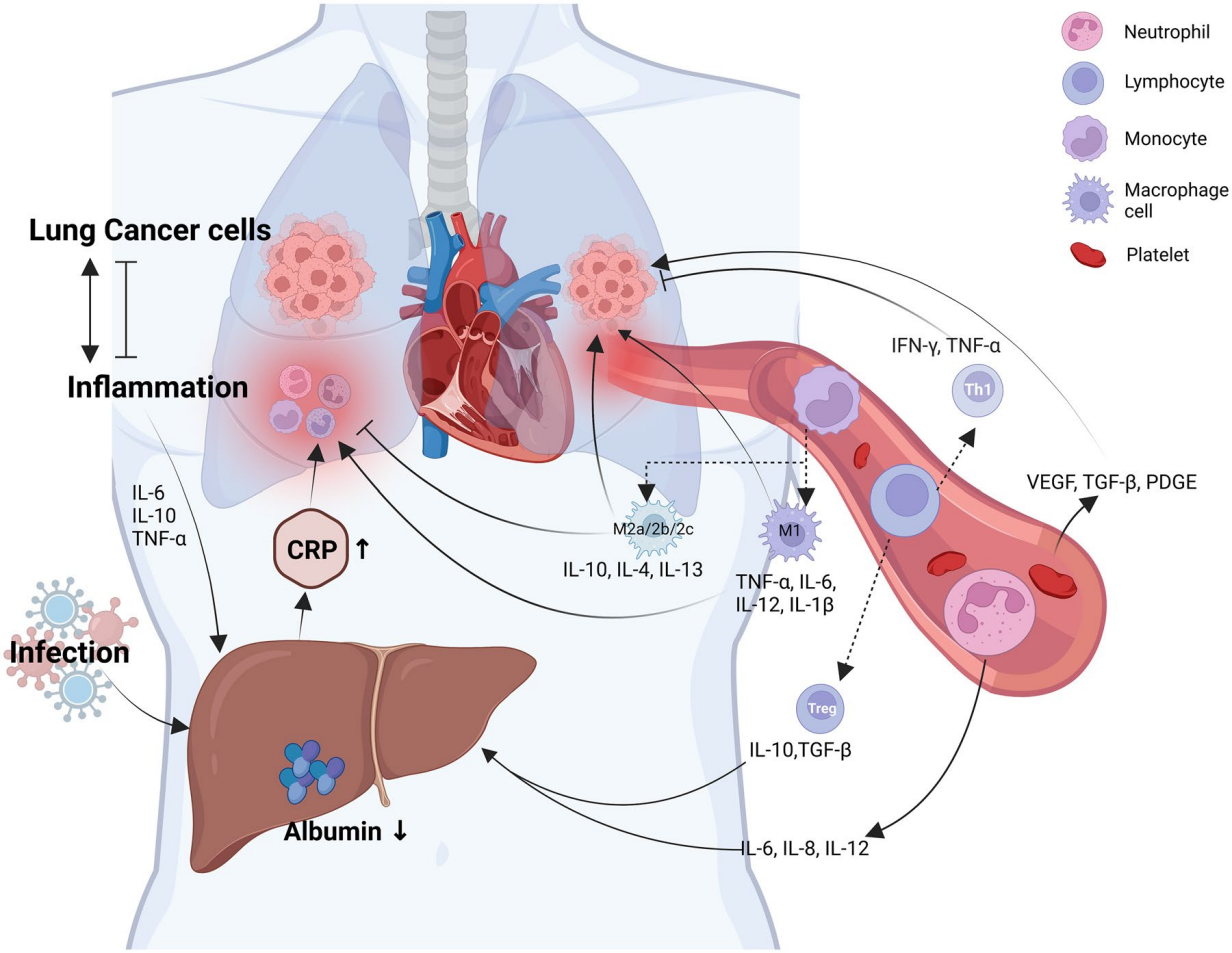
Pro-inflammatory activity of C-reactive protein and peripheral blood cells in non-small cell lung cancer patients with involvement of albumin. Figure created with BioRender.com. IL: interleukin; TNF: tumour necrosis factor; TGF: transforming growth factor; IFN: interferon; VEGF: vascular endothelial growth factor; PDGF: platelet-derived growth factor.

It is well-known that nutritional status affects the prognosis of NSCLC [10]. In patients with NSCLC, there is a complex interaction between Alb, CRP, and peripheral blood cells (Figure 1). As a biomarker, serum albumin (Alb) can not only reflect the nutritional status of the body but also remove pro-inflammatory stimulating factors in the body and relieve inflammatory reactions, indicating the level of systemic inflammatory status to a certain extent, which has a certain value in evaluating the prognosis of patients with NSCLC. And even studies have shown that its level can differentiate benign pulmonary nodules at the early stage of NSCLC [11]. Stares et al. [12] found that Alb < 35 g/L generally represented a poor prognosis for patients with metastatic non-small cell lung cancer (mNSCLC). The level of Alb can also predict the occurrence of adverse reactions in lung cancer patients after treatment [13]. A clinical study by Kazuki et al. showed that Alb was an independent predictor in NSCLC patients treated with programmed cell death protein‑1 (PD-1) inhibitors [14]. Gradually, it was realized that Alb was combined with some inflammatory markers, and a new composite marker was composed after a simple operation to evaluate the prognosis of patients with NSCLC. We call them ‘albumin-related inflammatory markers’. Clinical studies on the role of albumin-related inflammatory markers in the prognosis of NSCLC are in full swing. However, the current clinical application of these markers is not as enthusiastic as in the research. Clinicians do not fully understand the value of these markers and how they should be applied.

To systematically describe the role of these albumin-associated inflammatory markers in predicting the prognosis of NSCLC, this review synthesized the published clinical evidence on albumin-associated inflammatory markers in patients with NSCLC, reviewed their applications and cut-off values, and summarized their advantages and disadvantages. The findings may provide a useful reference for physicians on how to use the most appropriate prognostic tool in patients with NSCLC.

We explored the literature databases PubMed, EMBASE, Web of Science, and Cochrane Library for studies that may meet the criteria until January 2022. The search terms were set to ‘albumin’ and ‘inflammatory’ and ‘adenocarcinoma*’ or ‘Non-small cell lung cancer’ or ‘NSCLC’ or ‘LAD’ or ‘ADC’ and ‘Prognosis’. The population is limited to patients with a confirmed diagnosis of NSCLC histopathologically. A simple bibliometric visual analysis of the literature retrieved by PubMed was performed. For quantitative markers included in the review, we included them in the table of extracted information only when the literature accurately described its cut-off value and grouped them according to the high or low cut-off value. In this review, we analysed the prognostic value of albumin-related inflammatory markers based on CRP, such as CAR, GPS, MGPS, etc., and albumin-related inflammatory markers based on peripheral blood cells, such as PNI, ALI, etc.

## Bibliometric analysis of albumin and non-small cell lung cancer (2000–2021)

**Figure 2.**
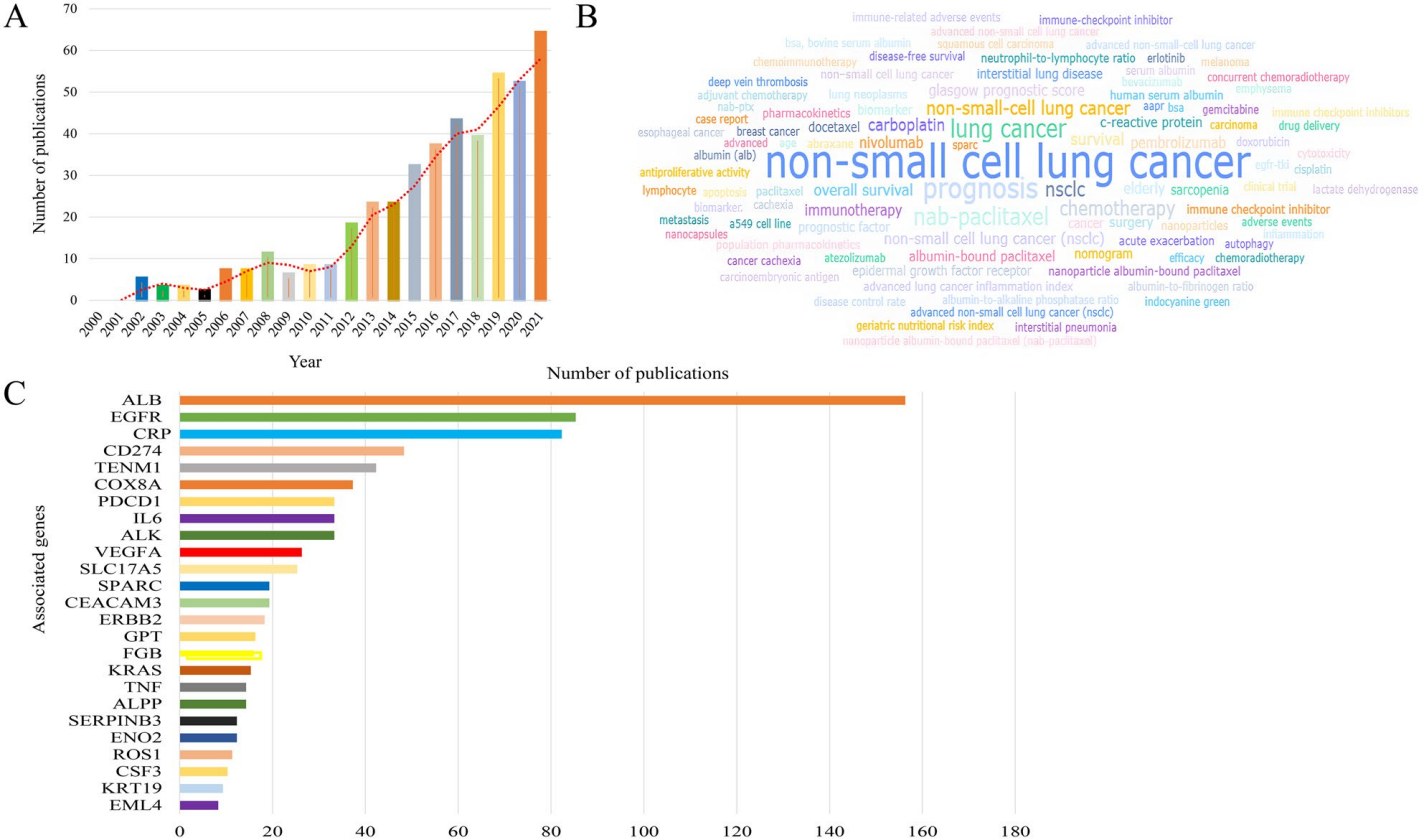
Bibliometric analysis of albumin and non-small cell lung cancer. (A) Number and trend of annual publications about albumin and non-small cell lung cancer. (B) Keyword frequency analysis of albumin and non-small cell lung cancer. (C) Analysis of associated genes of albumin and non-small cell lung cancer.

A total of 454 articles from 2000-01 to 2022-01 retrieved by PubMed were visualized by searching ‘albumin’ and ‘non-small cell lung cancer’ as keywords, and the average annual number of articles issued was 23. 2021 reached the peak of 64 annual documents, while the fastest growth rate of 250% was in 2006, suggesting that research in this field developed rapidly and was in a rapidly rising stage (Figure 2(A)). The keywords of the paper are to highly condense and summarize the research purpose, research object, and research method. Keyword-based analysis can reflect the trend of theme evolution and research hotspots within a certain period in a certain research field. As shown in Figure 2(B), the keywords in the top 5 occurrence frequencies were: ‘non-small cell lung cancer’, ‘prognosis’, ‘lung cancer’, ‘nab-paclitaxel’, and ‘NSCLC’. In the part of association gene analysis, we found that NSCLC was very closely related to ALB (Figure 2(C)).

## Albumin-related inflammation markers based on CRP

CRP is a typical acute-phase reaction protein. Its level rapidly increases during inflammation. It is considered to be one of the most widely used indicators of systemic inflammatory response. The C-reactive protein albumin ratio (CAR) was first proposed by Fairclough and colleagues [15]. Clinical studies in predicting the prognosis of lung cancer were first reported on small cell lung cancer (SCLC), and then gradually proved to have prognostic value in NSCLC [16,17]. In the beginning, ‘cumulative prognostic scores’ including CRP with Alb was proposed by Forest et al. [18]. The results showed that this score was more convenient for prediction in patients with NSCLC. In the following year, the Glasgow prognostic score (GPS) was first proposed and defined by Forest et al. which can also predict the prognosis [19]. Hypoalbuminaemia was not significantly associated with cancer-specific survival in the absence of elevated CRP concentrations. Therefore, patients with elevated CRP were assigned to a modified Glasgow prognostic score (mGPS) of 1 or 2 based on whether they had hypoproteinaemia [20,21]. These three markers are all based on a cut-off value of 10 mg/L for CRP and 35 g/L for Alb [22]. High-sensitivity modified Glasgow prognostic score (Hs-mGPS) was first applied to the study of NSCLC by Osugi et al. based on 3 mg/L for CRP [23]. Unlike Hs-mGPS, adjusted Glasgow prognostic score (a-GPS) raises the threshold for Alb (39 g/L) [24]. Albumin-related inflammatory markers based on CRP mainly include CAR, GPS, mGPS, Hs-mGPS, and a-GPS. Their definitions are detailed in Table 1.

**Table 1.** Description of the CAR, GPS, mGPS, Hs-mGPS, and a-GPS.

Full name	Abbreviation	Calculation formula	Reference
C-reactive protein albumin ratio	CAR	C-reactive protein/Albumin	[15]
Glasgow prognostic score	GPS	CRP ≤10 mg/L and albumin ≥35 g/L, score 0; CRP >10 mg/L or albumin <35 g/L, score 1; CRP >10 mg/L and albumin <35 g/L, score 2	[19]
Modified Glasgow prognostic score	mGPS	CRP ≤10 mg/L and albumin ≥35 g/L, score 0; CRP >10 mg/L and albumin ≥35 g/L, score 1; CRP >10 mg/L and albumin <35 g/L, score 2	[21]
High-sensitivity modified Glasgow prognostic score	Hs-mGPS	CRP ≤3 mg/L and albumin ≥35 g/L, score 0; CRP >3 mg/L and albumin ≥35 g/L, score 1; CRP >3 mg/L and albumin <35 g/L, score 2	[23]
Adjusted Glasgow prognostic score	a-GPS	CRP ≤3 mg/L and albumin ≥39 g/L, score 0; CRP >3 mg/L or albumin ≥39 g/L, score 1; CRP >3 mg/L and albumin <39 g/L, score 2	[24]

### CAR

The CRP/albumin ratio was proposed by Fairclough and his colleagues. But it was not defined as the abbreviation ‘CAR’ [15]. And its cut-off value was also vague. The first evidence-based medical report on NSCLC was performed by Miyazaki et al. [17]. In the article, they applied the abbreviation of ‘CAR’ and confirmed that preoperative CAR is a simple and objective indicator for predicting the prognosis of elderly patients with operable NSCLC. Unlike GPS, mGPS, and Hs-mGPS, CAR is quantitative. Therefore, we summarized the cut-off value of CAR (Table 2). From the table, it could be seen that there was a relatively confusing situation in the cut-off value of CAR, which may be the reason for the delay in the promotion. But it is undeniable that CAR, as a composite marker combining nutrition and inflammation, can be used as an indicator of whether surgical patients need to enhance nutrition and improve inflammation before surgery to a certain extent, with a good prospect of clinical application. In addition, it may also be helpful to recognize the possibility of early recurrence [4]. Matsubara et al. found that CAR could be used as the most valuable prognostic indicator of postoperative immunonutrition in patients with NSCLC [25].

**Table 2.** Summary of the characteristics of CAR in clinical studies.

Parameter	Participants’ conditions	cut-off value	Low/ High(N)	Outcome	AUC	Clinical findings	Reference
CAR	Elderly patients with operable NSCLC	0.28	59/49	OS	0.59	CAR is a prognostic marker, but GPS is not.	[17]
	Patients in pN2-stage IIIA with LADC	0.6	122/25	RFS	–	CAR is a prognostic marker, better than GPS, mGPS, Hs-mGPS, and PNI.	[30]
	Patients with operable NSCLC	0.424	492/125	DFS, OS	–	CAR is a prognostic marker.	[31]
	Patients with advanced NSCLC	0.2357	287/149	OS	0.700	CAR is a prognostic marker, better than GPS, mGPS, NLR, PLR and MLR.	[32]
	Chinese patients with NSCLC	0.14/0.22	148/110/129	OS	–	CAR is a prognostic marker, better than CRP, Alb.	[33]
	Patients with advanced NSCLC	0.35	38/39	OS	–	CAR may be a cheap, easy, and effective tool for predicting the death and its time of hospitalized NSCLC patients better than CRP.	[34]
	Patients with NSCLC underwent surgery	0.4	320/172	RFS	–	CAR and GPS may be independent risk factors for early recurrence.	[4]
	Patients with NSCLC underwent surgical resection	0.156	116/480	RFS, OS	0.587	CAR is a prognostic marker, better than GPS, mGPS.	[35]
	Patients with NSCLC treated with nivolumab	0.83	74/39	PFS, OS	–	CAR may be predictive of therapeutic response to nivolumab and long-term survival in NSCLC patients better than GPS, and NLR.	[36]

NSCLC: non-small cell lung cancer; LADC: lung adenocarcinoma; NLR: neutrophil-to-lymphocyte ratio; OS: overall survival; RFS: relapse-free survival; DFS: disease-free survival; PFS: progression-free survival; N: number of patients.

### GPS, mGPS, Hs-mGPS, and a-GPS

Studies on CAR let us realize that combining Alb with CRP may predict the prognosis of NSCLC, but it is calculated in the form of a ratio, which limits its application. Their ratios may not be very different when both Alb and CRP levels are high or low. Therefore, CAR as the ratio of CRP to Alb is not as objective as GPS and MGPS to some extent. When reviewing past clinical studies, the grouping of GPS is highly controversial, some are divided into 0 and 1 or 2 [26], and some are divided into 0, 1, and 2 [27]. Clinical studies on GPS have covered patients with stage I-IV NSCLC, and all of them have shown a good ability to predict prognosis. In our previous analysis, three groups of 0, 1, and 2 were performed according to the GPS. It was found that the GPS is an independent prognostic marker for patients with NSCLC regardless of the comparison between the two groups [28]. McMillan et al. found that the increase of CRP was often accompanied by a decrease of Alb [29]. The mGPS is then further improved based on a greater focus on the relationship between hypoproteinemia and cancer prognosis. Although only a few studies have made tentative adjustments to the cut-off value of CRP and Alb, along with the new prognostic markers of Hs-mGPS and a-GPS, it reminds us that more attention should be paid to the cut-off value of CRP and Alb [23,24].

## Albumin-related inflammation markers based on peripheral blood cells

Inflammation of the tumour microenvironment (TME) is characterized by the presence of host leukocytes in both stroma and tumour sites [37]. White blood cells include neutrophils and lymphocytes, eosinophils, basophils, and monocytes, with neutrophils and lymphocytes being the most strongly associated with inflammation [38]. Current studies have shown that neutrophils play a key role at different stages of tumour development. TME can influence the emergence of distinct neutrophil phenotypes that give rise to several key mediators associated with tumour growth and aggressiveness. The neutrophil-to-lymphocyte ratio (NLR) is a commonly used marker of systemic inflammation. NLR >5 is generally considered to indicate ongoing systemic inflammation [39]. NLR can be used to predict the prognosis of patients with stage IIIB-IV NSCLC treated with PD-1 inhibitors [40]. LMR has also been used as one of the markers of systemic inflammation [41,42]. In recent years, the role of platelet count in inflammation has also been gradually appreciated [43,44]. The emergence of the prognostic nutritional index (PNI) threatens the status of NLR to some extent [45,46]. Thus we describe albumin-related inflammatory markers based on peripheral blood cells, mainly including PNI, advanced lung cancer inflammation index (ALI), Alb concentration combined with NLR (COA-NLR), NLR × D-dimer count/albumin (NLDA), albumin and neutrophil combined prognostic grade (ANPG) and HALP. Their definitions were detailed in Table 3.

**Table 3.** Description of the PNI, ALI, COA-NLR, NLDA, ANPG, and HALP.

Full name	Abbreviation	Calculation formula	Cut-off value	Reference
Prognostic nutritional index	PNI	10 × albumin (g/dL) + 0.005 × absolute lymphocyte count (/μL)	48	[46]
Advanced lung cancer inflammation Index	ALI	(BMI × albumin) / NLR	18	[47]
Alb concentration combined with NLR	COA-NLR	NLR >2.5 or Alb <35 g/L, score 0; NLR >2.5 or Alb <35 g/L, score 1; NLR >2.5 and Alb <35 g/L, score 2	–	[48]
NLR × D-dimer count/Albumin	NLDA	NLR × D-dimer count/albumin	0.15	[49]
Albumin and neutrophil combined prognostic grade	ANPG	Grade 1 = elevated albumin and low neutrophil; Grade 2 = low albumin and low neutrophil, as well as elevated albumin and elevated neutrophil; Grade 3 = low albumin and elevated neutrophil.	albumin: 42.55 g/L; neutrophil: 2.895 × 10^9^/L	[50]
Haemoglobin, albumin, lymphocyte, and platelet score	HALP	haemoglobin (g/L) × albumin (g/L) × lymphocyte (/L)/platelet (/L)	48	[51]

NLR: neutrophil-to-lymphocyte ratio; BMI: body mass index.

### PNI

Kos et al. reported for the first time that the prognostic nutrition index (PNI) was applied to patients with NSCLC in clinical studies. It was found that PNI was a prognostic marker independent of other risk factors, with better ability than NLR in predicting mNSCLC [45]. As shown in Table 4, subsequent multiple studies obtained the predictive value of PNI in patients with completely resected NSCLC [52–58]. It also has prognostic value in elderly patients older than 75 years with NSCLC [53]. The acceptance of adjuvant chemotherapy, platinum-based chemotherapy, targeted therapy, immunotherapy, radiotherapy, and other different treatment modalities have good prognostic value in patients with NSCLC [59–68]. Through the difference between preoperative and postoperative values, the prognostic value of PNI in the perioperative period of NSCLC was confirmed [69,70]. Xu et al. reported for the first time that in patients with bone mNSCLC, higher PNI indicated a better prognosis [71]. Postoperative and advanced non-small cell lung cancer (aNSCLC) patients accounted for the majority of all included studies, which may mean that PNI has a higher prognostic value in these patients [72–76].

**Table 4.** Summary of the characteristics of PNI, ALI, NLDA, and HALP in clinical studies.

Parameter	Participants’ conditions	Cut-off value	Low/ High(N)	Outcome	AUC	Clinical findings	Reference
PNI	Patients with NSCLC	49.5	69/69	OS	–	PNI is an independent prognostic marker.	[45]
	Patients with completely resected NSCLC	50	149/241	OS	0.63	PNI is an independent prognostic marker.	[52]
	Elderly (aged > 75 years) patients with completely resected NSCLC	49.6	146/126	OS	0.532	PNI is an independent prognostic marker.	[53]
	Patients with NSCLC underwent radical surgery	52	912/504	OS	–	Higher PNI in NSCLC patients suggests a favourable prognosis.	[54]
	Patients with completely resected NSCLC	48	46/202	OS, RFS	–	PNI is an independent prognostic marker.	[55]
	Patients with completely resected NSCLC	45/50	57/134/324	OS	–	PNI is an independent prognostic marker.	[56]
	Patients with NSCLC underwent open thoracotomy for curative resection	50	285/726	OS, RFS	0.727	PNI was associated with postoperative pulmonary complications and long-term outcomes.	[57]
	Patients with NSCLC underwent surgical pulmonary resection and receive neoadjuvant therapy	46.810	37/73	OS, RFS	0.628	PNI is an independent prognostic marker.	[58]
	Patients with NSCLC received adjuvant chemotherapy	50	54/52	RFS	–	PNI is an independent prognostic marker.	[59]
	Patients with NSCLC treated with EGFR TKI	45	177/453	OS, PFS	–	Pre-treatment nutritional status is a prognostic marker.	[60]
	Patients with NSCLC treated with ICIs	45.5	52/50	OS, PFS	0.694	PNI is an independent prognostic marker.	[62]
	Patients with NSCLC underwent curative radiotherapy	45.45	–	OS	0.666	PNI is an independent prognostic marker.	[63]
	Patients with aNSCLC treated with PD-1 inhibitors	45	47/55	OS, PFS	–	PNI may be a useful predictive marker of clinical outcomes and irAEs.	[64]
	Patients with advanced NSCLC treated with platinum-based chemotherapeutics	52.525	54/45	OS, PFS	–	PNI is an independent prognostic marker.	[65]
	Patients with recurrence NSCLC after complete pulmonary resection who received ICI monotherapy during the therapeutic course	50	–	PFS	–	PNI is an independent prognostic marker.	[66]
	Patients with aNSCLC treated with PD-1 inhibitors	46.05	53/70	OS, PFS	0.780	PNI was an independent predictor of early progression and survival outcomes.	[67]
	Patients with metastatic or recurrent ALK-positive NSCLC received first-line alectinib	40	11/31	PFS	–	PNI is important in predicting, which reflect the nutritional status of the host	[68]
	Patients in stage IA-IIIB with NSCLC underwent chest surgery	47	193/282	OS, RFS	0.62	PNI has high predictive values for postoperative complications and survival.	[70]
	Patients with bone mNSCLC without any anti-tumor therapy	54.5	154/105	OS	–	PNI is an independent prognostic marker.	[71]
	patients in stage IIIB/IV with NSCLC	50	179/136	OS	–	PNI is an independent prognostic marker.	[72]
	Patients with aNSCLC	46.1	67/116	OS	0.55	Nutritional status is an important prognostic factor.	[73]
	Patients with mNSCLC treated with first-line chemotherapy	46.7	184/149	OS, PFS	0.617	PNI is an independent prognostic marker.	[74]
	Patients in stage IIIB with NSCLC	40.5	190/168	OS, PFS, LPFS	0.841	PNI is an independent prognostic marker.	[75]
	Patients with aNSCLC	38.4	52/108	OS, PFS	0.69	PNI is an independent prognostic marker.	[76]
ALI	Patients with mNSCLC	18	83/90	OS, PFS	0.67	ALI is an independent prognostic marker.	[47]
	Patients with mNSCLC	23.2	21/20	OS, PFS	–	ALI is an independent prognostic marker.	[78]
	Patients in stage IV with NSCLC	18	38/74	OS	–	ALI is an independent prognostic marker.	[79]
	Patients with aNSCLC initiated nivolumab treatment	18	69/128	PFS	–	ALI is an independent prognostic marker.	[80]
	Patients with mNSCLC received complete first-line treatment with chemotherapy	11	19/90	OS, PFS	0.52	ALI is associated with survival.	[81]
	Patients with NSCLC received complete resection	37.66	121/220	OS	0.681	ALI is an independent prognostic marker.	[82]
	Patients with NSCLC underwent VATS	41.20	125/214	OS	0.324	ALI is an independent prognostic marker.	[83]
	Patients in stage IA with NSCLC underwent lung resection	22.2	18/148	OS, RFS	0.610	ALI is an independent prognostic marker.	[85]
	Patients with early-stage NSCLC received VATS pulmonary resection as their only therapy	50	155/137	OS, DFS	–	ALI is an independent prognostic marker.	[86]
	Patients with NSCLC treated with nivolumab	18	–	PFS	–	A high ALI was predictive of better PFS in patients with poor performance status.	[87]
	Patients with mNSCLC	32.6	191/127	OS	–	ALI is an independent prognostic marker.	[88]
NLDA	IV stage NSCLC patients	0.15	32/12	OS	0.7	NLDA is an independent prognostic marker.	[49]
HALP	Patients with NSCLC underwent radical lung cancer resection	48	99/139	OS	0.666	HALP is an independent prognostic marker.	[51]

NSCLC: non-small cell lung cancer; aNSCLC: advanced non-small cell lung cancer; mNSCLC: metastatic non-small cell lung cancer; LA-NSCLC: locally advanced non-small cell lung cancer; TKI: tyrosine kinase inhibitor; PD-1: programmed cell death protein‑1; ICI: immune checkpoint inhibitor; irAE: immune-related adverse event; NLR: neutrophil-to-lymphocyte ratio; OS: overall survival; PFS: progression-free survival; LPFS: local progression-free survival; DFS: disease-free survival; RFS: relapse-free survival; VATS: video-assisted thoracic surgery; *N*: number of patients.

### ALI

The application of ALI was first reported in a clinical study of metastatic non-small cell lung cancer by Jafri et al. and the cut-off value was determined as 18 [47]. Alb and body mass index (BMI) are always used to determine nutritional status. According to the World Health Organization, patients are classified as underweight when their BMI is < 18.5 kg/m^2^. Therefore, ALI is a quantitative marker based on the numerical embodiment of ALB and BMI bilayer nutrition. We summarized the cut-off value of ALI (Table 4). Although the prognostic value of ALI was not found in the clinical studies by Kobayashi et al. and Watanabe et al. [4,77], multiple studies subsequently confirmed that lower ALI tended to predict poor prognosis in patients with NSCLC [78,79]. Whether receiving targeted treatment [80], 1st line chemotherapy [81], radical surgery [82,83], or chemotherapy combined with targeted treatment [84], ALI shows prognostic value. Even in patients at the early stage of NSCLC, ALI also has a prognostic value [85,86]. In lung cancer patients with poor performance status, ALI has a more prominent prognostic value [87,88]. Palomar-Abril et al. noted that the prognostic role of ALI in NSCLC was not affected by age [89]. Furthermore, it is exciting that Mountzios et al. confirmed that ALI lost its predictive ability when chemotherapy was added to immunotherapy by dividing patients into Cohort ‘A’ (PD-L1 inhibitors in any treatment line alone), cohort ‘B’ (first-line chemo-immunotherapy), and cohort ‘C’ (platinum-based first-line chemotherapy). ALI is a powerful prognostic and predictive marker for the efficacy of immunotherapy when immune checkpoint inhibitors (ICIs) are used as a single therapy rather than in combination with chemotherapy. This also illustrates that for patients with PD-L1-high, an ALI >18 may help select patients who do not require additional chemotherapy [90]. Tomita et al. pointed out that the combined detection of ALI and CRP was a useful indicator for predicting overall survival, and could be used as a simple prognostic tool to help identify patients with operable NSCLC [91].

### Others

In addition to PNI, and ALI, two widely studied albumin-related inflammatory markers based on peripheral blood cells, there are other less-studied markers. Weng and colleagues’ clinical studies proposed a new effective biomarker for prognosis in NSCLC patients treated with resection value. Preoperative COA-NLR can effectively stratify prognosis in NSCLC patients by classifying patients into three independent groups [48]. An innovative attempt was made by Sun et al. which put forward a new prognostic marker–NLDA [49]. In terms of this marker, the role of the D-dimer count was also added, and coagulation factors were considered. A retrospective study of 272 patients with stage IV NSCLC showed that NLDA was an independent adverse prognostic factor [49]. The albumin and neutrophil combined prognostic grade (ANPG) was proposed by Sun et al. It was confirmed that higher ANPG independently predicted OS and PFS in patients with NSCLC [50]. HALP is a more comprehensive score that reflects the nutritional status of patients through haemoglobin and albumin and the inflammatory status of patients through LMR. The results of clinical studies confirmed that HALP was an independent prognostic marker [51].

## Discussion

Both systemic inflammatory response and poor nutritional status are widely recognized as risk factors for poor prognosis in patients with NSCLC. The combination of the two may be able to make up for their limitations. From the results of bibliometric analysis, literature related to albumin and non-small cell lung cancer show an increasing trend. The heat of keywords such as ‘prognosis’ and ‘CRP’ enlightens us that the inflammation index related to albumin, as a kind of low-cost and less invasive predictive test method, has shown great research value in recent years. Albumin-related inflammation markers are increasingly being evaluated to enhance the prediction of prognosis in NSCLC. More importantly, Alb, CRP, and peripheral blood cells are standardized tests that are easily performed and obtained in daily work. We summarized and compared the advantages, disadvantages, and range of cut-off value of these markers. At the same time, their suitable patients are compared (Table 5). However, most of them only appear in clinical research rather than clinical practice. The main reason for this is that there is no comprehensive assessment of its prognostic role which is specific and sensitive. Its cut-off value is uncertain, and there is little validation of stable prediction models. Therefore, for the identification of predictive markers, improvement and appropriate use are of great interest. Predicting the development of NSCLC and survival after treatment will enable us to better select patients and improve the utilization of expensive resources. And it will be the key to saving healthcare resources.

**Table 5.** Comparison of advantages and disadvantages of albumin-related inflammatory markers, its suitable patients, and range of cut-off value.

Albumin-related inflammatory markers	Advantages	Disadvantages	Suitable patients	Range of cut-off value
CAR	An effective tool for predicting the survival of NSCLC patients.	The cut-off value is controversial.	Operable elderly patients with NSCLC.	0.14–0.83
GPS, mGPS, Hs-mGPS, a-GPS	More objective than CAR.	Inflexible. Lack of research on prognostic value in NSCLC patients after being treated with immunotherapy.	NSCLC patients, whose CRP and Alb values do not fluctuate around normal values.	–
PNI	A sensitive tool for predicting the survival of NSCLC patients.	Poor specificity.	Postoperative or advanced NSCLC patients.	38.4–52.525
ALI	The prognostic value is not affected by age.	Poor sensitivity and specificity.	Patients with aNSCLC or treated with immunotherapy.	11–50
COA-NLR	Preoperative COA-NLR can effectively stratify the prognosis of patients.	Inflexible. There are few related studies.	Patients with NSCLC underwent lung resection.	–
NLDA	Coagulation factors are considered.	There are few related studies.	Patients with NSCLC in the IV stage.	0.15
HALP	It is less likely to change under the influence of one factor.	There are few related studies.	Patients with NSCLC underwent lung resection.	48

NSCLC: non-small cell lung cancer; aNSCLC: advanced non-small cell lung cancer; CAR: C-reactive protein albumin ratio; GPS: Glasgow prognostic score; mGPS: modified Glasgow prognostic score; Hs-mGPS: high-sensitivity modified Glasgow prognostic score; a-GPS: adjusted Glasgow prognostic score; PNI: prognostic nutritional index; ALI: advanced lung cancer inflammation Index; COA-NLR: Alb concentration combined with NLR; NLDA: NLR × D-dimer count/Albumin; ANPG: albumin and neutrophil combined prognostic grade; HALP: haemoglobin, albumin, lymphocyte and platelet score.

**Figure 3.**
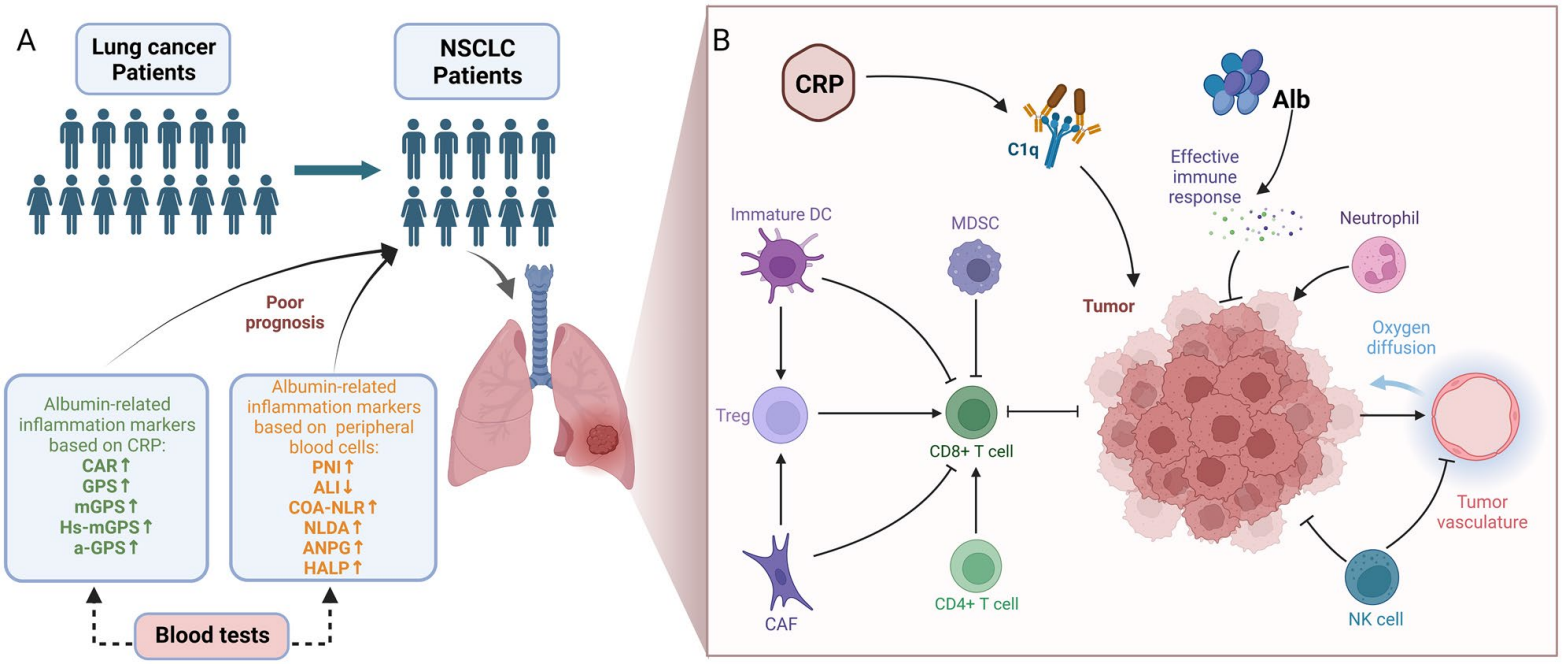
Conceptual framework for albumin-related inflammatory markers in the prognosis and immune microenvironment of non-small cell lung cancer. (A) Increased or decreased albumin-related inflammatory markers predict poor prognosis in patients with non-small cell lung cancer. (B) Albumin, C-reactive protein, and peripheral blood cells play a role in the immune microenvironment of NSCLC. Figure created with BioRender.com. CAR: C-reactive protein albumin ratio; GPS: Glasgow prognostic score; mGPS: modified Glasgow prognostic score; Hs-mGPS: high-sensitivity modified Glasgow prognostic score; a-GPS: adjusted Glasgow prognostic score; PNI: Prognostic nutritional index; ALI: Advanced lung cancer inflammation Index; COA-NLR: Alb concentration combined with NLR; NLDA: NLR × D-dimer count/albumin; ANPG: albumin and neutrophil combined prognostic grade; HALP: haemoglobin: albumin: lymphocyte and platelet score; DC: dendritic cell; MDSC: myeloid-derived suppressor cell; CAF: cancer-associated fibroblast; NK: natural killer.

To the best of our knowledge, this is the first review of current albumin-related inflammation markers. In this review, albumin-related inflammation markers based on CRP or peripheral blood cells were introduced, including CAR, GPS, mGPS, HS-mGPS, a-GPS, PNI, ALI, COA-NLR, NLDA, ANPG, and HALP, to predict the prognosis of patients with NSCLC **(**Figure 3(A)). The key to the role that these markers can play in predicting the prognosis of non-small cell lung cancer may be due to the role of Alb, CRP, and peripheral blood cells in the immune microenvironment of non-small cell lung cancer. The main reason for low Alb in oncology patients is the specific inhibition of Alb gene transcription by TNF [92], which impairs the antitumor immune response activated by Alb. The different roles played by each CRP isoform at sites of local inflammation and infection [93], but what is certain is that CRP is an immune regulator, not just a marker of inflammation or infection, activating C1q to promote lung cancer progression. Similarly, peripheral blood cells play an important role in the NSCLC microenvironment. Neutrophils secrete IL-6, IL-8, and IL-12 to promote the tumour inflammatory microenvironment, and the proliferating tumour cells in turn stimulate neutrophils, forming a vicious circle [94,95]. Lymphocytes are the main immune cells in the body and play a key role in immune surveillance by inhibiting the proliferation, invasion and migration of tumour cells. Monocytes are precursors of macrophages and are also tropic for tumours and their inflammatory microenvironment, promoting tumour invasion [96]. Platelets secrete transforming growth factor-β (TGF-β), vascular endothelial growth factor (VEGF) and platelet derived growth factor(PDGF), which play a role in tumour progression and metastasis [97] (Figure 3(B)). Obviously, there is more room for optimization of albumin-related inflammation markers. Significantly, the optimal cut-off value of quantitative markers is confusing. This confusion is mainly due to differences in statistical analysis and different clinicopathological characteristics of patients with NSCLC. However, this still suggests that in subsequent clinical studies, attention should be paid to the cut-off value of quantitative markers such as CAR, PNI, ALI, etc., and if the receiver operating characteristic curve (ROC) is applied, the area under curve (AUC) should be reported. In addition, we believe that there is a need for clarity regarding the time points at which these markers are monitored. For example, values monitored before treatment, values monitored after treatment, or the difference between before and after treatment are worth being compared.

Anti-PD-1 monotherapy reduces T-cell apoptosis and improves neutrophil and monocyte function. It has shown promising results in NSCLC treatment [98]. The increase in immunotherapy has increased the 5-year survival rate of NSCLC patients from 5% to 26% [99]. Along with the use of immunotherapy, markers to predict prognostic risk and drug response in NSCLC patients receiving immunotherapy have been sought. Tumor mutational load (TMB) [100], epidermal growth factor receptor (EGFR) mutations [101], and soluble programmed cell death ligand-1 (sPD-L1) [98] can predict response to immunotherapy. However, their detection is cumbersome and expensive. We observed that higher CAR in patients with NSCLC treated with nivolumab predicted poorer treatment response [36]. PNI can predict OS and PFS in patients receiving immunotherapy [62,64,66]. ALI has prognostic value in predicting survival in patients with NSCLC treated with nivolumab [87]. Other than that, other markers have not been seen in immunotherapy studies. The prognostic value of these markers in terms of efficacy response to immunotherapy is an area that deserves deeper investigation.

It has to be admitted that the nutritional status reflected by albumin has a limited effect on the prognosis of patients with NSCLC. As a result, the clinical value of albumin-related inflammation markers becomes limited. The Controlling nutritional status (CONUT) based on the serum levels of albumin, cholesterol, and lymphocyte count has been shown to predict the efficacy and prognosis of NSCLC patients treated with pembrolizumab [102]. With the addition of cholesterol, it may be more valuable than the albumin-related inflammation markers described in the article. In addition, it is not difficult to find that some of the ‘participants’ conditions’ we have included have experienced surgery. Therefore, these albumin-related inflammation markers may show greater clinical value in predicting the prognosis of patients with NSCLC undergoing surgery. But at the same time, we must note that patients with NSCLC who can undergo surgery generally have no dietary restrictions, and supplemental nutrition alone is not expected to dramatically improve nutritional indices, let al.one improve the prognosis of patients. And for those patients with poor nutritional indicators but in the early stage of NSCLC, there may still be a good prognosis, so it is necessary to adjust important confounding factors and conduct a long-term follow-up prospective cohort study. Before the cut-off value is determined, it is difficult for these markers to achieve the transformation from research to clinical application. In addition, finding people who are suitable for predicting prognosis, personalizing and accurate, and developing more intelligent inflammation prediction or diagnostic markers related to albumin may be a necessary step to realize them from clinical study to clinical application.

Our review also had some limitations. In the literature we included, single-centre retrospective studies accounted for the majority. And in our review of the included studies, we focused on the value of albumin-related inflammatory markers and the main findings of the study, without too much consideration of the methods of these studies and their limitations. There is also a bias that negative results are not published and cannot be included in the study. Moreover, in addition to albumin-related inflammatory markers, there are many other combined inflammatory markers and scores that can predict the prognosis of NSCLC. Reports on the prognostic value of SIS have never stopped [103]. The predictive value of scores or markers combining CRP with BMI, NLR, and LYM in patients with non-small cell lung cancer has also been reported [104–106]. Recently, a novel tumour marker and inflammation index (TMII) based on serum CEA and CRP has been reported. High preoperative TMII predicted a poor prognosis in patients with NSCLC [107]. But they have not been systematically reviewed in our article.

## Conclusion and perspectives

There is no clinical study comparing the current albumin-related inflammatory markers. The calculation of these markers is similar and has a similar predictive effect in previous studies. This review provided in-depth thinking on how to better study and use these markers. It is necessary to adjust important confounding factors and conduct a long-term follow-up prospective cohort study to further clarify their cut-off value and respective application advantages. In addition, the specific mechanism of how these markers affect the prognosis is not clear, which also encourages us to further study it.

## Data Availability

All data were included in the manuscript and there was no restriction on availability.
